# The *Porphyromonas gingivalis* inhibitory effects, antioxidant effects and the safety of a Sri Lankan traditional betel quid - an in vitro study

**DOI:** 10.1186/s12906-020-03048-6

**Published:** 2020-08-20

**Authors:** Madhavi Priyanka Paranagama, Nadisha Sewwandi Piyarathne, Tharanga Lakmali Nandasena, Sumedha Jayatilake, Ayanthi Navaratne, Bandula Prasanna Galhena, Senani Williams, Jayantha Rajapakse, Kiyoshi Kita

**Affiliations:** 1 Department of Basic Sciences, Faculty of Dental Sciences, University of Peradeniya, Peradeniya, Sri Lanka; 2 Department of Oral Medicine and Periodontology, Faculty of Dental Sciences, University of Peradeniya, Peradeniya, Sri Lanka; 3 Department of Chemistry, Faculty of Science, University of Peradeniya, Peradeniya, Sri Lanka; 4 Department of Biochemistry and Clinical Chemistry, Faculty of Medicine, University of Kelaniya, Ragama, Sri Lanka; 5 Department of Pathology, Faculty of Medicine, University of Kelaniya, Ragama, Sri Lanka; 6 Department of Pathobiology, Faculty of Veterinary Medicine & Animal Science, University of Peradeniya, Peradeniya, Sri Lanka; 7 School of Tropical Medicine and Global Health, Nagasaki University, 1-12-4, Sakamoto, Nagasaki, 852-8523 Japan

**Keywords:** Sri Lankan traditional betel quid, Chronic periodontitis, *P. gingivalis*, Oxidative stress, Genotoxicity, Cytotoxicity, MTT assay, CBMN assay

## Abstract

**Background:**

The Sri Lankan traditional betel quid (TBQ) which had been extensively used in the country before its colonization is claimed to have antiperiodontopathic effects in the Sri Lankan folklore. However, there is no reported scientific evidence to support the claimed antiperiodontopathic effects mediated by this TBQ. The present study was carried out to investigate the protective effect of the Sri Lankan TBQ in the pathogenesis of periodontitis.

**Methods:**

We investigate the ethyl acetate extract of the Sri Lankan TBQ for its antibacterial effects against the keystone periodontopathic bacterium, *P. gingivalis* and also its antioxidant potential, which is important to protect the periodontium from oxidative stress. Further, its safety was analyzed using the cytokinesis block micronucleus assay on human peripheral blood lymphocytes (PBLs).

**Results:**

Ethyl acetate extract of this TBQ inhibited the growth of *P. gingivalis* with a minimum bactericidal concentration (MBC) of 125 μg/ml. It was found to be a rich source of polyphenols and displayed considerable DPPH and ABTS radical scavenging activities and a strong ferric reducing antioxidant power. This extract could protect the cultured human gingival fibroblasts from H_2_O_2_ induced oxidative stress. In addition, this TBQ extract was not genotoxic to human PBLs even at a concentration of 2.5 mg/ml. Moreover, it exhibited protective effects against bleomycin induced genotoxicity in PBLs.

**Conclusion:**

Ethyl acetate extract of the Sri Lankan TBQ is a source of natural antibacterial compounds against *P. gingivalis*. It is also a source of natural antioxidants which can protect human gingival fibroblasts from H_2_O_2_ induced oxidative stress. These properties of the TBQ may have contributed to its claimed antiperiodontopathic effects. Besides, it was found to be relatively non-toxic to human cells. Thus this TBQ extract has a huge potential to be developed as a novel adjunctive therapeutic lead against periodontitis.

## Background

Periodontitis, which is identified as the sixth most common infectious disease in the world, is characterized by destruction of the periodontal tissues including gingiva, periodontal ligament and the alveolar bone with eventual loss of teeth [1, 2]. Several lines of evidence indicate that periodontitis not only affects the oral health, but also the general health of an individual since it is associated with other systemic conditions such as cardiovascular diseases, diabetes mellitus, rheumatoid arthritis, respiratory diseases, and preterm low birth weights [3–7]. Thus, this disease imposes a strong negative impact on the overall wellbeing of the affected individuals and the economy of a country.

Periodontitis is caused by periodontopathic bacteria in the subgingival biofilms [8]. The gram negative anaerobic bacterium *Porphyromonas gingivalis* is identified as the keystone bacterium in the development of this disease [9, 10]. However, the presence of periodontopathic bacteria alone is not sufficient for development of the disease. A complex interplay between the periodontopathic bacteria and the host immune system is essential for the initiation and progression of periodontitis [11, 12]. In response to periodontopathic bacteria, the innate immune system of the host induces a strong local inflammatory response, which is characterized by an intense recruitment of polymorphonuclear leukocytes to the periodontal tissues [13]. These cells produce massive amounts of superoxide radicals (O_2_•−) which are subsequently released into the extracellular environment and converted into a multitude of radical and non-radical derivatives, such as hydrogen peroxide (H_2_O_2_), hypochlorous acid (HOCl), hydroxyl radical (OH•) and singlet oxygen (^1^O_2_). These reactive oxygen species (ROS) are known to play a significant role in the pathogenesis of periodontitis through direct destruction of bio-molecules and signalling the release of inflammatory mediators [14, 15]. Therefore, any antiperiodontopathic medicine used in prophylaxis or treatment of periodontitis must have antibacterial effects against the periodontopathic bacteria or/and cytoprotective effects against ROS to minimize periodontal tissue destruction.

The time tested Sri Lankan TBQ containing *Piper betle* (leaves), *Syzygium aromaticum* (flower buds), *Myristica fragrans* (seeds and mace), *Elettaria cardamomum* (fruits), *Areca catechu* (nuts), *Kaempferia galanga* (rhizomes) and *Coriandrum sativum* (seeds), which has been chewed after the three major meals (approximately 3 g of fresh betel leaves and 2 g of other ingredients in dried form) is claimed to be effective against periodontitis in the Sri Lankan folklore. However, there was no scientific evidence on these attributes of the Sri Lankan TBQ. Indeed, the scientific literature provides some evidence for the in vitro *P. gingivalis* inhibitory effects of the individual ingredients in the TBQ [16–22], but the final antibacterial effect of a herbal mixture cannot be calculated by adding their individual antibacterial effects since synergistic and antagonistic interactions among them also contribute to the final outcome [23]. The antioxidant effects analyzed in cell free systems also have been reported for the individual ingredients [24–30], but their cytoprotective effects depend on the bioavailability of them in the relevant tissues [31, 32]. Thus, this study was undertaken to investigate the potential antibacterial effects of the TBQ against the keystone periodontopathic bacterium, *P. gingivalis* and its potential antioxidant effects against oxidative stress induced damage to fibroblasts; the principal cell type in the periodontium. In addition, the safety of the TBQ extract was investigated using the cytokinesis block micronucleus (CBMN) assay on human PBLs.

## Methods

### Plant materials

Leaves of *Piper betle* were obtained from the betel research institute, Narammala, Sri Lanka. Seeds of *Areca catechu*, fruits of *Elettaria cardamomum*, Seeds and mace of *Myristica fragrans*, flower buds of *Syzygium aromaticum*, and rhizomes of *Kaempferia galanga* were obtained from local spice growers in the Central Province, Sri Lanka. Seeds of *Coriandrum sativum* were purchased from a local indigenous herbal product vendor. All plant ingredients were authenticated by the botanist at the national herbarium, Royal Botanical Gardens, Peradeniya, Sri Lanka. Their voucher specimens (MP2017001-MP2017003 and MP2018001-MP2018004) were deposited at the same herbarium (Table 1).

**Table 1:** Common names, botanical names and the morphological parts of the constituent medicinal plants of the TBQ

Common name	Botanical name	Morphological part of the plant	Classification number
Clove	*Syzygium aromaticum* (L.) Merr. & Perry	Flower buds	MP2017001
Cardamom	*Elettaria cardamomum* (L.) Maton	Fruits	MP2017002
Coriander	*Coriandrum sativum* L.	Seeds	MP2017003
Betel	*Piper betle* L.	Leaves	MP2018001
Areca nut	*Areca catechu* L.	Seeds	MP2018002
Nutmeg- seed	*Myristica fragrans* Houtt.	Seeds & Seed coverings	MP2018003
Java galangal	*Kaempferia galanga* L.	Rhizomes	MP2018004

### Preparation of the TBQ extract

Fresh samples of the herbal ingredients were washed and air dried. Afterwards, they were ground into a fine powder and mixed in equal portions. Ten grams of this mixture were extracted in 100 ml of ethyl acetate (EA). Extractions were done in a soxhlet apparatus for 2 h at 30 °C and the extracts were concentrated under reduced pressure in a rotary evaporator and lyophilized. The yield of the dried extract was 30.5 ± 1.9%. w/w (n = 3). The dried extracts were stored at −20 °C and reconstituted in dimethyl sulfoxide (DMSO) and filter sterilized using 0.2 μm nylon filters before analyses.

### Chemicals and equipment

Ethyl acetate and H_2_O_2_ were purchased from BDH, UK. dimethyl sulfoxide (DMSO), 1`1` diphenyl 2 picrylhydrazyl (DPPH), ferric chloride, ferrous sulphate, methanol, 2,4,6-triphyridyl-s-triazine (TPTZ), and 2,2-azinobis-(3-ethylbenzothiazolin-6-sulfonic acid (ABTS), potassium persulfate, ascorbic acid, gallic acid, trolox and 3-(4,5-dimethylthiazole-2-yl)-2,5-diphenyl-etrazoliumbromide (MTT) were purchased from Sigma-Aldrich Inc. (USA). Dulbecco’s modified eagle’s medium (DMEM), Rosswell Park Memorial Institute (RPMI) 1640 medium, foetal bovine serum (FBS), penicillin/streptomycin, glutamine, phytohaemagglutinin and cytochalasin B were purchased from Gibco BRL (USA). Bleomycin (BLM) was purchased from United Biotech, India. Brain heart infusion (BHI) broth, BHI agar, blood agar and anaerobic sachets were from Oxoid (UK). Defibrinated sheep blood was from the Veterinary Research Institute, Gannoruwa, Sri Lanka. UV-1800 UV-Vis spectrophotometer, Shimadzu, Japan and Multiskan Ex plate reader from Thermoscientific, USA, were used for antioxidant and MTT assays respectively.

### Bacteria

*P. gingivalis* (ATCC 33277 ) was obtained from the American Type Culture Collection and revived on blood agar supplemented with 5% sheep blood, hemin (5 mg/ml) and vitamin K1 (0.5 μg/ml) under anaerobic conditions for 7 d at 37 °C. After verifying the colony morphology and bacterial morphology after Gram staining, the bacterium was inoculated to BHI broth containing hemin (5 mg/ml) and vitamin K1 (0.5 μg/ml) and incubated under anaerobic conditions for 3 d. *P. gingivalis* stock cultures prepared from this broth culture were maintained in 40% v/v glycerol at -80 °C throughout the study period.

### Antibacterial effects of the TBQ extract against *P. gingivalis* using agar well diffusion assay

EA extract dissolved in 10% DMSO was tested for dose dependant antimicrobial effects using agar well diffusion assay as described by Madduluri et al. [33] and Gamboa et al. [34]. The stock culture of the bacterium was grown on sheep blood agar supplemented with 5% sheep blood, hemin (5 mg/ml) and vitamin K1 (0.5 μg/ml) for 7 d under anaerobic conditions. After verifying the colony morphology of cultured bacteria, 3–5 colonies were cultured in BHI broth supplemented with hemin (5 mg/ml) and vitamin K1 (0.5 μg/ml) under anaerobic conditions for 72 h. This culture of *P. gingivalis* was adjusted to 0.5 McFarland standard; 1.5 × 10^8^ Colony Forming Units (CFU/ml) and streaked on the surface of agar using a sterile swab. Then 9 mm wells were made on agar plates using a stainless steel borer. Afterwards, the wells were filled with 200 μl of the extract at concentrations of 7.5, 15 and 30 mg/ml. Gentamycin (0.1 mg/ml) and 0.2% chlorhexidine were used as positive controls and 10% DMSO was used as a negative control. After incubating the plates for 72 h, bacterial colonies became visible on the surface of the agar plates except in the areas of growth inhibition around the wells. It took 5 d for the bacteria to develop black pigments. The zones of inhibition were measured using a micrometer gauge. Extracts which showed clear zones around the wells were considered to have an inhibitory effect on the bacterium and those which did not show clear zones without bacterial growth were considered to have no inhibitory effect on the bacterium.

### Antibacterial effects of the TBQ extract against *P. gingivalis* using minimum bactericidal concentration (MBC)

The MBC was determined by standard broth micro dilution assay according to CLSI guidelines [35]. Briefly, 2-fold serial dilutions of the extracts were prepared and added to BHI broth supplemented with haemin and vitamin K1. The final concentrations of the extract in the wells ranged from 62.5 μg/ml - 8 mg/ml. The microtitre plate wells were inoculated with an inoculum of 10^5^ CFU and incubated at 37 °C for 72 h under anaerobic conditions. Afterwards, 2 μl of each culture was spotted on to a fresh solid medium and incubated for 5 d under same conditions. The lowest concentration that yielded no bacterial growth on solid medium was taken as the MBC.

### Total phenolic content (TPC)

TPC of the TBQ extract was measured as described by Singleton and Rossi [36]. First, the dried extract was dissolved in DMSO to obtain a 0.5 mg/ml solution. Then, a 200 μl of sample was mixed with 1 ml of 1:10 diluted Folin Ciocalteu reagent, and incubated at room temperature for 10 min. Afterwards, 800 μl of 7.5% (w/v) solution of Na_2_CO_3_ was added and incubated for further 30 min at room temperature. Absorbance was measured at 743 nm. Gallic acid standards in the range of 20–100 μg/ml were used for preparation of the standard curve. Results are expressed as mg of gallic acid equivalents/g of dry weight (DW) of the extract.

### DPPH radical scavenging activity

DPPH radical scavenging activity of the TBQ extract was measured as described by Williams et al. [37]. For each sample 5 different concentrations (12.5, 25, 50, 75, and 100 μg/ml) were tested. Briefly, 200 μl of the sample (0.5 mg/ml) diluted in DMSO was added to 1.8 ml of 1 mM DPPH-methanol solution. The samples were incubated for 30 min at room temperature and the absorbance was read at 517 nm. DPPH-methanol solution with 200 μl of DMSO was used as the blank control. Inhibition percentage for each concentration was calculated using the following equation.

$$ \mathrm{Percentage}\ \mathrm{inhibition}=\frac{\mathrm{Absorbance}\ \mathrm{of}\ \mathrm{the}\ \mathrm{blank}-\mathrm{Absorbance}\ \mathrm{of}\ \mathrm{the}\ \mathrm{sample}}{\mathrm{Absorbance}\ \mathrm{of}\ \mathrm{the}\ \mathrm{blank}}\mathrm{x}100 $$

For each sample, percentage inhibition was plotted against the concentration and IC_50,_ which is defined as the amount of antioxidant required to inhibit the initial DPPH concentration by 50% was calculated. A higher DPPH radical scavenging activity is reflected by a lower IC_50_ value.

### Trolox equivalent antioxidant capacity (TEAC)

TEAC of the TBQ extract was analyzed as described by Rei et al. [38]. For this assay, ABTS radical cation (ABTS^•+^) was produced by reacting a 7 mM ABTS stock solution with 2.45 mM potassium persulfate (final concentration) and incubating the mixture at room temperature in the dark for 16 h. The ABTS^•+^ solution was diluted with methanol to obtain an absorbance of 0.70 at 734 nm and the assay was carried out by mixing 10 μl of the sample (1 mg/ml) with 1 ml of reagent and incubating it for 6 min at 30 °C. Trolox, a water soluble vitamin E analogue was used as the standard and the results are expressed as mg of trolox equivalents /g of DW of the extract.

### Ferric reducing antioxidant power (FRAP)

FRAP of the TBQ extract was estimated by the method of Benzie and Strain [39]. The FRAP reagent was freshly prepared by mixing 10 mM TPTZ solution in 40 mM HCl, 20 mM FeCl_3_, and 300 mM acetate buffer (pH 3.6) in 1:1:10 (v/v/v) proportions. Afterwards, 20 μl of the sample (0.2 mg/ml) was mixed with 1 ml of the reagent, incubated for 4 min at room temperature and observed the absorbance at 593 nm. FeSO_4_ solutions in the concentration range of 200–1000 μM were used to plot the standard curve. The FRAP was expressed as millimoles of ferrous equivalents/g of DW of the extract.

### Assessment of the cytotoxicity of the TBQ extract

To identify a suitable concentration of this extract to be applied in cytoprotective assays, it was initially screened for cytotoxic effects using MTT assay as described by Illeperuma et al. [40]. For this purpose, human gingival fibroblasts, HGF-1 (ATCC-CRL 2014) obtained from the American Type Culture Collection (ATCC, Manassas, VA, USA), were cultured in DMEM–F12 medium supplemented with, 10% heat-inactivated FBS, 2 mM L-glutamine, penicillin (100 IU/ml) and streptomycin (100 μg/ml) at a density of 2 × 10^4^ cells/well in 96 well cell culture plates (Corning) and incubated in a humidified, 5% CO_2_ incubator at 37 °C. Afterwards, cells were treated with the TBQ extract serially diluted in DMEM containing 1% DMSO as the vehicle for 1, 3 and 5 days. BLM at a concentration of 100 μg/ml served as the positive control and DMEM containing 1% DMSO served as the negative control. After treatment, the cells were rinsed three times with phosphate buffered saline (PBS) pH 7.4, and the MTT assay was performed as described in Methods in Molecular Biology [41]. The absorbance was measured at 570 nm and 620 nm and the data were normalized to the negative control.

### Assessment of the cytoprotective effect of the TBQ extract

Cytoprotective effect of the tested extract against H_2_O_2_ induced cytotoxicity on HGF-1 cells was tested according to the method used by Sazwi et al. [42]. Based on the cytotoxicity assay results, a dose of 100 μg/ml of the TBQ extract was selected as a suitable dose for the assessment of cytoprotectivity. For this assay also, the cells were seeded and incubated as described above. When the cells became 70% confluent, the medium was aspirated and replaced with new medium containing 100 μg/ml of the TBQ extract and incubated for another 24 h. Subsequently, the cells were rinsed three times with PBS and treated with different concentrations of H_2_O_2_ (0–2 mM) for 6 h under standard conditions. The control samples were treated with medium only. Finally, the cells were rinsed with PBS three times and the MTT assay was performed as described elsewhere [41]. The data were normalized to negative control.

### Assessment of the safety of the TBQ extract

Safety of the TBQ extract for human use was analyzed using Cytokinesis Block Micronucleus (CBMN) assay, which is recommended by the Food and Drug Administration, USA for testing pharmaceuticals intended for human use [43, 44]. Briefly, peripheral venous blood was collected into heparinized tubes from 4 healthy voluntary donors (two males and two females; 30–50 yr who were non-smoking, non-betel quid chewing and with no recent history of radiation exposure) after obtaining informed written consent. For each individual, five peripheral blood lymphocyte (PBL) cultures were set up, each containing 500 μl of whole blood and 4.5 ml of RPMI 1640 culture medium supplemented with 10% FBS, 200 mM L-glutamine, penicillin (100 units/ml) and streptomycin (100 mg/μl). First culture served as the negative control and was added with 1% DMSO. Second culture served as the positive control and added with 40 μg/ml BLM. Third and fourth cultures which served as TBQ genotoxicity tests were added with 250 μg/ml and 2.5 mg/ml of TBQ extract respectively. Fifth culture which served as TBQ antigenotoxicity test was added with 40 μg/ml BLM after preincubating the cells in 2.5 μg/ml TBQ extract for 3 h. All cultures were incubated for 3 h at 37 °C in a humidified CO_2_ incubator. Subsequently, cells in each culture were washed with PBS, re-suspended in 4.5 ml of complete medium, added with 5 μg/ml of phytohaemagglutinin, and incubated for another 44 h. Then, cytokinesis was arrested by adding 3 μg/ml of cytochalasin-B to the culture. After another 28 h, the cells were harvested with a brief hypotonic treatment and fixed with Carney’s fixative (methanol:acetic acid - 3:1). Thereafter, cell suspensions were dropped on to clean glass slides, air dried and stained with Giemsa stain. All the slides were coded and scored blindly. Thousand binucleated lymphocytes with a preserved cytoplasm were counted and the frequency of micronuclei (MN) was expressed as a percentage.

### Statistical analysis

For antibacterial assays, antioxidant assays, cytotoxicity assays and cytoprotective assays, three independent extracts (*n* = 3) were analyzed on three different days and the results are expressed as mean ± standard deviation (SD). For CBMN assay, the micronuclei were counted in 1000 binucleated cells from 4 independent cultures (*n* = 4) and the MN frequency is expressed as a percentage. The sample means were compared by ANOVA followed by post hoc Tukey’s test. In addition, for the cytoprotective assay, Student t test was also used to compare the means of TBQ treated and untreated groups. *P* < 0.05 was considered significant. Data were analyzed using Graph Pad Prism software (version 6, USA).

## Results

### Antibacterial effect of the TBQ extract against *P. gingivalis*

**Fig. 1:**
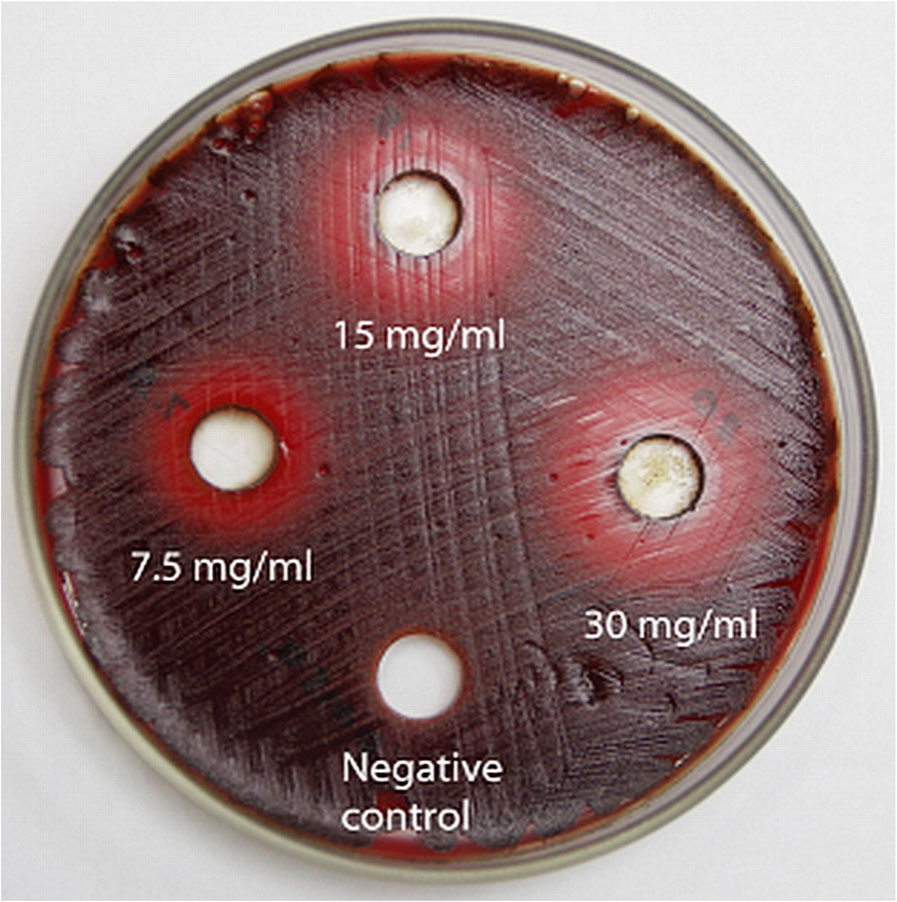
A photograph of a bacterial culture plate showing the dose dependent growth inhibitory effects of the TBQ extract on *P.gingivalis*. The clear zones around the wells indicate the zone of inhibition

Initial screening of the TBQ extract using agar well diffusion assay revealed that it has inhibitory effects against *P. gingivalis*. As shown in Fig. 1, the zones of inhibition at concentrations of 7.5, 15 and 30 mg/ml of the extract against this bacterium were, (14.33 ± 1.15) mm, (16.33 ± 2.08) mm and (17.50 ± 1.80) mm respectively while the negative control (10% DMSO) had no detectable inhibition. The zones of inhibition for the positive controls (gentamycin and chlorhexidine) were 24.0 ± 1.2 mm and 18.0 ± 0 mm respectively. When the same extract was tested using broth micro dilution method, it showed a MBC of 125 ± 0 μg/ml.

### TPC and antioxidant potential of the extract

Next we analyzed this TBQ extract for its TPC since plant polyphenols are known to be the major phytochemical responsible for their antioxidant effects. As shown in Table 2, TBQ extract has a TPC of 114.0 ± 1.3 mg of gallic acid equivalents/g of DW. The IC_50_ value for its DPPH radical scavenging activity was 20.6 ± 1.8 μg/ml while the ABTS radical scavenging activity was 624.8 ± 8.5 mg of trolox equivalents/g of DW. Its FRAP was found to be 3688.9 ± 223.8 mmol/g of DW.

**Table 2:** Antioxidant potential and the TPC of the EA extract of the Sri Lankan TBQ

Extract/Positive control	DPPH radical scavenging activity (IC_50_: μg/ml)	ABTS radical scavenging activity (mg of trolox equivalents /g of DW)	Ferric reducing antioxidant power (mmol of ferrous equivalents /g of DW)	Phenolic content (mg of gallic acid equivalents /g of DW)
TBQ extract	20.6 ± 1.8	624.8 ± 8.5	3688.9 ± 223.8	114.0 ± 1.3
Ascorbic acid*	4.2 ± 0.1	1263.8 ± 39.0	8068.4 ± 62.3	–
Trolox*	6.3 ± 0.1	–	5176.8 ± 33.0	–

The results are expressed as mean ± SD of 3 independent experiments. * indicates standard antioxidants.

### Cytotoxicity of the extract

**Fig. 2:**
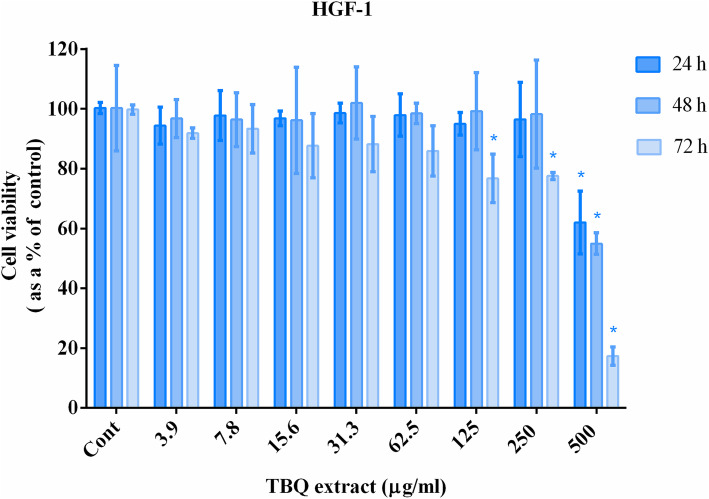
Time and dose dependant effects of the TBQ extract on the viability of HGF-1 cells. The bars represent the mean of 3 independent experiments (*n* = 3) and the error bars represent their standard deviation. The mean of the data denoted by * is significantly different from that of the corresponding negative control. The level of significance was *P* < 0.05

Before applying the TBQ extract to cell cultures for analyzing its cytoprotective effects on gingival fibroblasts, we analyzed its cytotoxicity using the MTT assay. As shown in Fig. 2, even a dose of 250 μg/ml of the TBQ extract did not display a significant cytotoxic effect on cultured human gingival fibroblasts after 24 and 48 h. Even after 72 h, the TBQ extract did not show significant cytotoxic effect at doses < 125 μg/ml. However, cytotoxic effects were detected after 72 h, when the cells were exposed to > 125 μg/ml of the extract. At the highest dose of the TBQ extract tested in this study (500 μg/ml), cytotoxic effects could be detected even after 24 h. Therefore, it was decided to expose the HGF-1 cells to this extract at a dose of 100 μg/ml for 24 h when analyzing its cytoprotective effect against oxidative stress.

### Cytoprotective effect of the extract

**Fig. 3:**
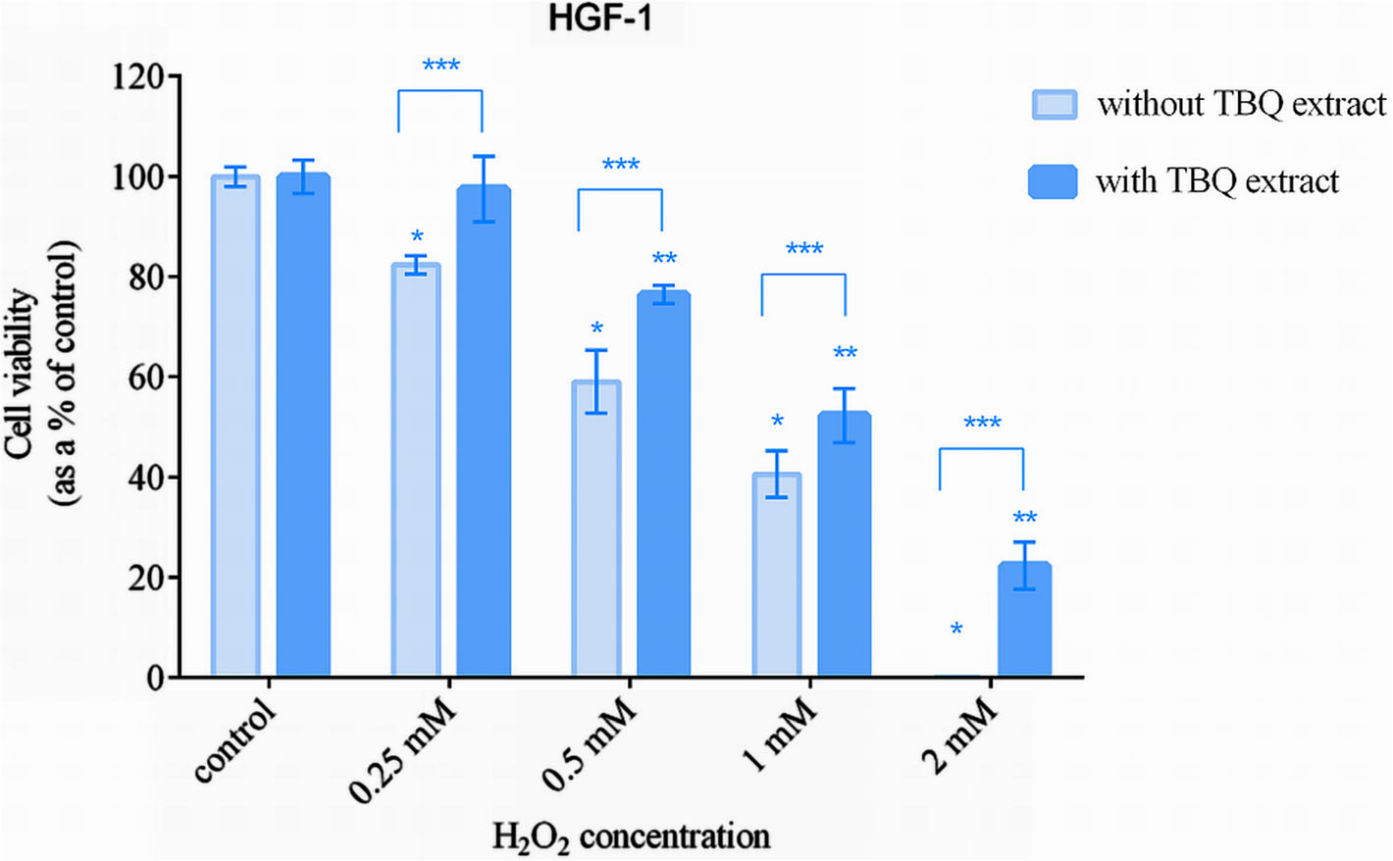
Dose dependent effects of H_2_O_2_ on the viability of HGF-1 cells with and without pretreating with the TBQ extract at a dose of 100 μg/ml for 24 h. The bars represent the mean of 3 independent experiments (n = 3) and the error bars represent their standard deviation. The data denoted by * and ** are significantly different from the corresponding negative control. The data denoted by *** is significantly different from that of the test without TBQ extract. The level of significance was P < 0.05

Next, we attempted to see whether the potent antioxidant activities detected in the TBQ extract were capable of protecting human gingival fibroblasts from oxidative stress induced cytotoxicity. This was tested by challenging the gingival fibroblasts with H_2_O_2_ after preincubating the cells with the TBQ extract at a dose of 100 μg/ml for 24 h. As shown in Fig. 3, the cells treated with H_2_O_2_ for 6 h exhibited a significant reduction in cell viability when compared with the control cells. However, for each tested dose of H_2_O_2_, the reduction in viability of the cells pretreated with the TBQ extract was significantly lower than that of the cells which were not pre-treated with the TBQ extract.

### Safety of the TBQ extract

**Fig. 4:**
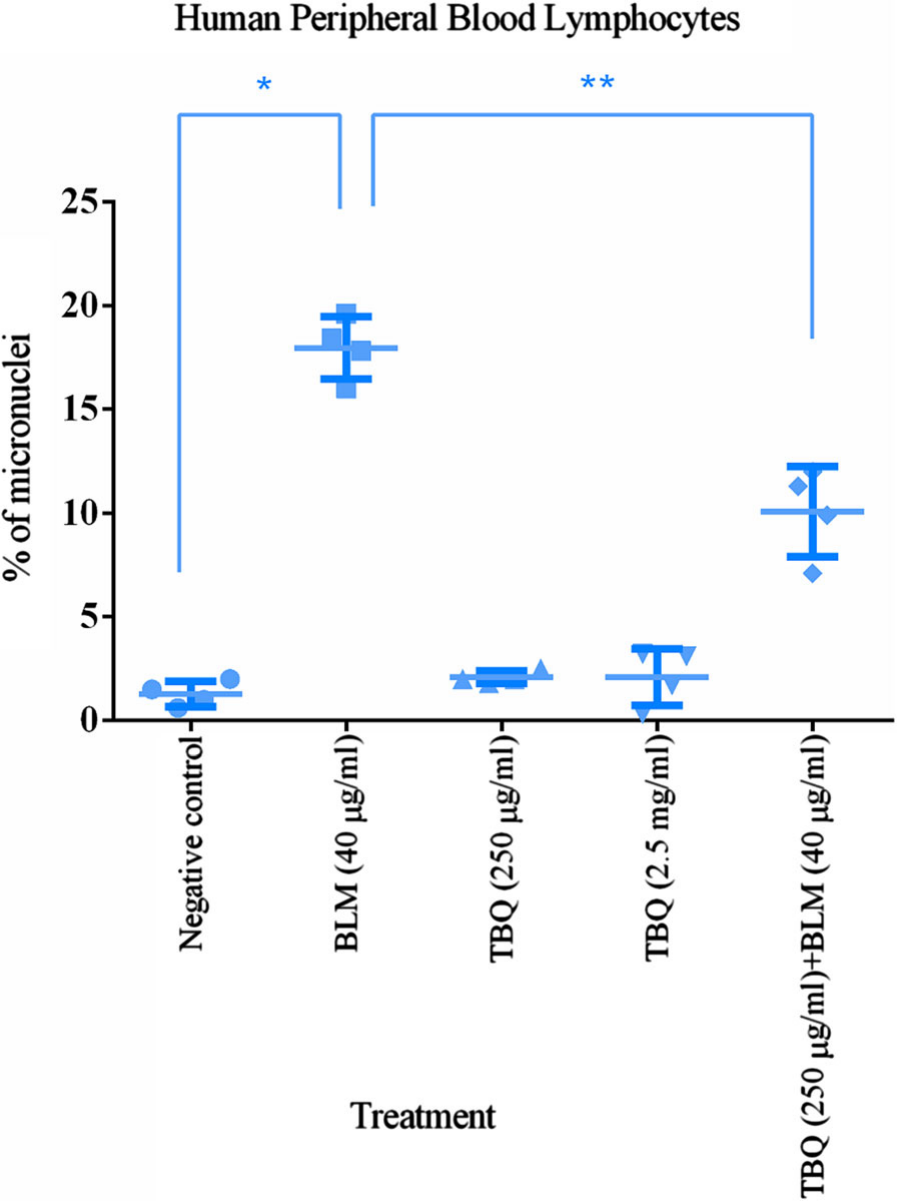
CBMN assay data, expressed as the % of micronuclei in 1000 binucleated lymphocytes for 4 individuals in each treatment group with mean and SD. The data denoted by * and ** are significantly different from the BLM treated group. The level of significance was P < 0.05

Finally, we explored the safety of the TBQ extract using the CBMN assay. As shown in Fig. 4, the mean MN frequency in the negative control group (0.1% DMSO) was 1.28 ± 0.61%. The MN frequencies of the TBQ treated group at concentrations of 250 μg/ml and 2.5 mg/ml were 2.08 ± 0.30% and 2.08 ± 1.37% respectively. These MN frequencies were not significantly different from that of the negative control group. In contrast, the MN frequency of the positive control (BLM treated) group was significantly higher than that of the negative control group (18.85 ± 0.90%). Interestingly, BLM induced MN formation was significantly reduced (10.08 ± 2.17%) when BLM was added after pre-treating the cells with the TBQ extract for 3 h.

## Discussion

Periodontitis is a multifactorial disease. The primary cause of periodontitis is infection of periodontal tissues with periodontopathic bacteria. Among them, *P. gingivalis* has been identified as the keystone bacterium. In the present study, we first show that the EA extract of the Sri Lankan TBQ is a rich source of natural antibacterial compounds against *P. gingivalis* with a MBC value of 125 μg/ml. It provides clear evidence for the *P. gingivalis* inhibitory effects of the Sri Lankan TBQ, which must be partly responsible for its claimed antiperiodontopathic effects.

When we compared the *P. gingivalis* inhibitory effects of the TBQ extract tested in this study with those reported for its individual ingredients, it was higher in the TBQ extract than that of most of its individual ingredient extracts. For example, EA extract of *M. fragrans* seeds and methanol extract of *K. galanga* were reported to have MBCs of 250, 320 μg/ml respectively [20, 21] while methanol extract of *S. aromaticum* was reported to have a MIC of 625 μg/ml [18]. Besides, the MBC of *M. fragrans* mace has been reported to be > 640 μg/ml [20]. Even though MIC and MBC values were not reported for crude extracts of *P. betle* and *A. catechu*, certain phytochemicals isolated from these ingredients were reported to have *P. gingivalis* inhibitory effects. For example, one of the major polyphenols in *P. betle* (hydroxychavicol) was reported to have a MBC between 62.5–500 μg/ml [16] and tannic acid, one of the major polyphenols in *A. catechu*, was reported to have a ZOI of 2 mm against *P. gingivalis* using disc diffusion assay [17]. On the other hand, essential oils from coriander leaves were reported to have a MBC of 125 μg/ml [22].

Moreover, the *P. gingivalis* inhibitory effect of the tested Sri Lankan TBQ extract was higher than that of many other herbal extracts reported to have antiperiodontopathic effects. Even the MICs of the water extract of *Polygonum tinctorium Lour* (MIC:1.74 mg/ml), methanol extract of *Terminalia laxiflora* (MIC:0.25 mg/ml), methanol extracts of *Ambrosia maritima* (MIC:0.5 mg/ml), seeds of *Argemone mexicana* (MIC: 0.5 mg/ml), wood and bark of *Terminalia brownii* (MIC: 0.5 mg/ml), bark of *Combretum hartmannianum* (MIC: 0.5 mg/ml), bark of *Acacia tortilis* (MIC:0.5 mg/ml) and the ethanol extract of *Piper marginatum* Jacq (MIC: 1 mg/ml), ethanol extract of *Ilex guayusa* Loes (MIC: 1 mg/ml) methanol extract of *Phytolacca americana* (MIC: 600 μg/ml), water extract of *Camellia sinensis/*green tea (MIC: 12.5 mg/ml), ethanolic extract of *Allium sativum/*garlic (MIC: 62.5 mg/ml), ethanolic extract of *Mammea americana* (MIC: 500 μg/ml), and the water extract of *Castanopsis lamontii* (MIC: 625 μg/ml) were higher than the MBC of the TBQ extract against *P. gingivalis* [34, 45–51]. According to Kohlie et al., ethanol extract of the husk of *Coccus nucifera* also has a lower *P. gingivalis* inhibitory effect (MBC:1562.5 μg/ml) compared to that of the tested TBQ extract [52].

Another factor which has been previously demonstrated to have a significant contribution to the pathogenesis of periodontitis is oxidative stress. It has been found that exogenous antioxidants can be used to counteract the tissue oxidative stress by boosting the endogenous antioxidant defence mechanisms. Specially, plant polyphenols have been known to play a key role in this process [53]. According to our results, the Sri Lankan TBQ has a TPC of 114.0 ± 1.3 mg of GAE /g of DW showing it to be a rich source of polyphenols.

The plant polyphenols are known to neutralize free radicals through numerous mechanisms [54]. One of them is donation of hydrogen ions to neutralize their unpaired electrons/anionic radicals. When the hydrogen ion donating potential of the TBQ extract was assessed using the DPPH radical scavenging assay, it was found to have an IC_50_ value of 20.6 ± 1.8 μg/ml, indicating its strong hydrogen ion donating ability. When this IC_50_ value was compared with that of purified form of ascorbic acid, the antioxidant potential of the TBQ extract was found to be approximately 20% that of ascorbic acid (IC_50_ for ascorbic acid: 4.2 ± 0.1 μg/ml). Another mechanism of plant polyphenols in reducing oxidative stress is through donation of electrons. When the electron donating ability of the TBQ extract was measured using ABTS^**.**+^ radical scavenging assay with trolox as a standard, it showed a TEAC of 624.8 ± 8.5 mg/g of DW, indicating that the free radical scavenging activity of this extract is comparable to approximately 50% that of ascorbic acid (1263.8 ± 39.0 mg/g). In addition, this extract showed a considerable FRAP also (3688.9 ± 223.8 mmol/g of DW), indicating its ability to serve as a potent antioxidant through donation of electrons. Its FRAP was 40% that of ascorbic acid (8068.4 ± 62.3 mmol/g of DW). Collectively, these findings provide scientific evidence to prove that this Sri Lankan TBQ extract is a rich source of natural antioxidants.

However, previous studies have shown that cytoprotective effects of plant derived antioxidants depend not only on their potency but also on their bioavailability in the cells. Therefore, if the antioxidant molecules in the TBQ have a cytoprotective effect, they must be readily available to the most abundant population of cells in the periodontium; the fibroblasts. Providing evidence for the bioavailability of these antioxidants to these cells, H_2_O_2_ induced cytotoxicity in cultured human gingival fibroblasts were attenuated when these cells were preincubated with the TBQ extract. Previous studies have shown that plant derived antioxidants can reduce the oxidative stress in human cells through scavenging of free radicals or induction of antioxidant enzymes [55, 56]. Thus, the protective effect of the TBQ extract against oxidative stress observed in the current study may be attributed to the ability of natural antioxidants in this extract to directly neutralize H_2_O_2_ molecules entering the cells or indirectly neutralize them through induction of intracellular antioxidant enzymes such as catalases and peroxidases.

The antioxidant properties we have reported for the TBQ extract in this study are comparable with results of similar studies on its individual ingredients reported in the literature. When the DPPH assay results were taken into consideration, methanol extract of *P. betle* from Sri Lanka was reported to have a IC_50_ of 12.66 ± 0.07 μg/ml and a water extract of a Malaysian variety of *A. catechu* was reported to have a IC_50_ of 7.5 ± 0.5 μg/ml [24, 25]. According to these values, the DPPH radical scavenging activities of *P. betle* and *A. catechu* are higher than that of the TBQ extract tested in this study. In contrast, the IC_50_ for the ethanol extract of *S. aromaticum* (42 ± 7.4 μg/ml), methanol extracts of Tunisian, Syrian and Egyptian varieties of *C. sativum* (27.00 ± 6.57 μg/ml, 36.00 ± 3.22 μg/ml and 32.00 ± 2.87 μg/ml), ethanol extract of *E. cardamomum* (217.43 μg/ml), mace and seed of *M. fragrans* (49.78 ± 2.76 μg/ml and 115.13 ± 0.53 μg/ml) were higher than that of the TBQ extract, providing evidence for their lower DPPH radical scavenging activities [29, 57–59]. *K. galanga* also is reported to be a rich source of antioxidants, but its DPPH radical scavenging activity cannot be compared with the results of this study since it was expressed as ascorbic acid equivalents (17 ± 1 mg AA/100 g) [60].

Finally, the safety of this TBQ extract was analyzed using the CBMN assay on cultured human PBLs, since it is a pre requisite to analyze the safety of any herbal medicine intended for human use. According to our results, this TBQ extract was not genotoxic at the highest tested dose (2.5 mg/ml). Moreover, pretreatment of the PBLs with the TBQ extract could protect these cells from BLM induced genotoxicity. This finding provides scientific evidence for the antigenotoxic potential of the TBQ extract. It is a well established fact that BLM induced genotoxicity is free radical mediated [61]. Thus, neutralization of BLM induced free radicals by the antioxidants in the TBQ extract is one of the possible mechanisms for the observed antigenotoxic effects in this study. The other possible mechanism may be induction of DNA repair enzymes contributing to repair of the DNA double strand breaks before micronuclei formation. The antigenotoxic effects shown for the TBQ extract in this study are comparable to the results of previous studies which have shown the ability of phytochemicals to neutralize the genotoxic effects of BLM [62, 63].

Our study provides first scientific evidence for the ability of the Sri Lankan TBQ extract to act on two etiological factors contributing to the pathogenesis of periodontitis. It clearly indicates the potential of this extract to be used in multitargeted therapy in prevention and treatment of periodontitis. Cytoprotective and antigenotoxic effects detected in this study are added advantages of this herbal mixture. It is also noteworthy that the extremely low cytotoxic and gentotoxic effects as well as the cytoprotective and antigenotoxic effects we have reported for the TBQ in this study are in contrast to the cytotoxic and genotoxic effects reported for the contemporary betel quid (CBQ) used in Sri Lanka and the other south Asian countries [64, 65].

Eventhough there are geographical variations, the major ingredients in the CBQ are betel, arecanut (> 50% of the dry weight) and slaked lime with or without tobacco [66, 67]. Both arecanut and tobacco in this mixture have carcinogenic potential [68]. Slaked lime in this mixture can enhance the carcinogenic potential of CBQ by creating an alkaline environment in the oral cavity and thereby enhancing the release of carcinogenic alkaloids from arecanut and generation of free radicals from those alkaloids. In addition, slaked lime can cause ulceration of the oral cavity and expose the basal layer of the oral mucosa for mutagenic compounds [69, 70]. In contrast, the TBQ tested in this study does not contain tobacco or slaked lime. However, it contains a relatively low content of arecanut (12.5% of the mixture) when compared to the CBQ. Both epidemiological and animal studies have shown an association between arecanut and development of oral potentially malignant disorders (OPMDs) and the risk of oral cancer [68, 71, 72]. However, it has been demonstrated that such association depends on the frequency and the dose of arecanut consumption [71, 73]. Despite this claimed potential carcinogenic effect, small doses of arecanut have been used alone or in combination with other medicinal herbs in treating diverse disease conditions in Asian traditional medicine systems [74–76]. A number of recent laboratory and clinical studies have also provided strong evidence for the therapeutic effect of low concentrations of arecoline, which is known to be the carcinogenic alkaloid in arecanut. According to Cheng et al. a low concentration of arecoline (< 100 μg/ml) can induce death of human hepatoma cells in a targeted manner with minimal effects on normal hepatocytes [77]. The anticancer effect mediated by low doses of arecoline has been further supported by both in-vitro and in-vivo studies using cultured human cancer cell lines and in nude mice bearing tumour xenografts [78]. Moreover, in clinical studies, multiple doses of arecoline have been reported to improve the cognitive function of Alzheimer patients [79]. In a schizophrenic mouse model, arecoline was found to alleviate the memory impairment and demyelination of neurons [80]. The multiple target approach in herbal medicine primarily focusses on maximizing the therapeutic outcome mediated by multiple compounds on vast array of targets, perhaps promoting the synergism and neutralising toxic effects of each individual compound. One of the important findings related to alleviation of arecanut toxicity is co-administration antioxidants. Investigations by Illeperuma et al. have shown that antioxidant rich herbal extracts can prevent arecanut induced ROS generation and genotoxicity in immortalized human oral keratinocytes and DNA double strand breaks in a mouse model [81]. Investigations by Zhou et al. have shown that vitamins C and E can alleviate hepatoxic effects of arecanut in mice [82]. These findings support the use of arecanut in combination with other antioxidant rich herbal ingredients in traditional medicine systems, thus alleviating any possible detrimental effects [74]. In the present study, the content of arecanut accounts only for 1/8th of the TBQ mixture, while the remaining 7/8th portion is shared by seven herbal ingredients which are rich sources of natural antioxidants [24, 26–30]. Moreover, these herbal ingredients are reported to possess anticancer effects as well [83–88]. Thus, the presence of proportionately low content of arecanut along with seven other herbal ingredients rich in antioxidants and anticancer compounds and the absence of tobacco and slaked lime in the tested TBQ justifies the observed low cytotoxic and genotoxic effects. It shows the potential of this herbal mixture to be used as a substitute for the currently used CBQ also. However, further detailed studies are suggestive to quantify the arecoline content of this TBQ extract and optimize its ingredients to obtain the maximum therapeutic effect while minimizing the toxic effects. Some of the limitations of our study are the lack of data on the antibacterial effects of TBQ extract against other periodontopathic bacteria and the clinical isolates of *P. gingivalis*. Our future studies are directed towards overcoming these limitations. Since the current trend in addressing chronic inflammatory diseases such as periodontitis is multi-targeted therapy, further in vitro and in vivo tests are warranted to understand other targets of the TBQ in the pathogenesis of periodontitis and also to develop oral health care products using this herbal mixture to control periodontitis.

## Conclusion

Overall outcome of this study supports the use of the EA extract of the Sri Lankan TBQ as a combined therapy for prevention and treatment of periodontitis due to its antibacterial, antioxidant, cytoprotective and non-genotoxic effects. Thus this formulation could be developed as a promising natural mouth rinse or a periodontal gel with multiple actions for the management of gingival diseases.

## Data Availability

The datasets analysed during the current study are available from the corresponding author on reasonable request.

## Abbreviations

ABTS: 2,2-azinobis-3-ethylbenzothiazolin-6-sulfonic acid
BHI: Brain Heart Infusion
BLM: Bleomycin
CBMN: Cytokinesis block micronucleus assay
CBQ: Contemporary Betel Quid
CFU: Colony Forming Units
CLSI: Clinical and Laboratory Standard Institute
DMSO: Dimethyl sulfoxide
DMEM: Dulbecco’s Modified Eagle’s Medium
DPPH: 1`1` diphenyl 2 picryl hydrazyl
DW: Dry weight
EA: Ethyl acetate
FRAP: Ferric reducing antioxidant power
GAE: Gallic acid equivalents
HGF-1: Human Gingival Fibroblasts-1
MBC: Minimum Bactericidal concentration
MN: Micronuclei
MTT: 3-(4,5-dimethylthiazole-2-yl)-2,5-diphenyl-etrazoliumbromide
OPMDs: Oral Potentially Malignant Disorders
PBLs: Peripheral Blood Lymphocytes
PBS: Phosphate Buffered Saline
RPMI: Rosswell Park Memorial Institute
TBQ: Traditional Betel Quid
TEAC: Trolox equivalent antioxidant capacity
TPC: Total phenolic content
TPTZ: 2,4,6-triphyridyl-s-triazine
